# Retroperitoneal Extra‐Adrenal Paraganglioma Presenting With Secondary Amenorrhea and Hypertension in an Adolescent: A Rare Case From Sub‐Saharan Africa

**DOI:** 10.1002/ccr3.73034

**Published:** 2026-06-22

**Authors:** Getasew Kassaw Alemu, Ibraist Yohannes Haileyesus, Hirsi Haahi Ahmed, Ahmednasir Abdi Hasan, Mubarek Abdi Mohamed

**Affiliations:** ^1^ Department of Internal Medicine University of Gondar, College of Medicine and Health Sciences Gondar Ethiopia; ^2^ Jigjiga University, College of Medicine and Health Sciences Jigjiga Ethiopia; ^3^ Addis Ababa University, College of Health Sciences Addis Ababa Ethiopia

**Keywords:** amenorrhea, extra‐adrenal paraganglioma, hypertension, retroperitoneal neoplasms, Sub‐Saharan Africa

## Abstract

Extra‐adrenal paragangliomas are rare neuroendocrine tumors that may cause hypertension and reproductive dysfunction in adolescents. We report a 16‐year‐old girl with uncontrolled hypertension and secondary amenorrhea caused by a functional Organ of Zuckerkandl paraganglioma. Surgical excision restored normal blood pressure and menstruation, highlighting reversible catecholamine‐induced hypothalamic–pituitary–gonadal axis suppression.

## 1 Introduction

Pheochromocytomas and paragangliomas (PPGLs) are rare neuroendocrine tumors arising from chromaffin cells of the adrenal medulla or extra‐adrenal neural crest–derived cells, respectively [1, 2]. Approximately 80% are adrenal pheochromocytomas, while 20% are extra‐adrenal paragangliomas [2]. Patients typically present with the classic triad of episodic headache, palpitations, and sweating, often accompanied by sustained or paroxysmal hypertension [2].

Paragangliomas are classified based on their origin: parasympathetic tumors are usually nonfunctional and located in the head and neck, whereas sympathetic paragangliomas arise along the thoracoabdominal chain and are frequently catecholamine‐secreting [2, 3]. The Organ of Zuckerkandl, located near the origin of the inferior mesenteric artery, is a recognized site for extra‐adrenal sympathetic paragangliomas [3, 4].

PPGLs are uncommon in pediatric populations, accounting for less than 20% of cases [5]. Endocrine manifestations such as secondary amenorrhea are particularly rare [6]. Up to 30%–40% of cases are associated with hereditary syndromes involving genes such as SDH, VHL, RET, and NF1, emphasizing the importance of genetic evaluation [7].

Chronic catecholamine excess can suppress the hypothalamic–pituitary–gonadal axis, resulting in reversible hypogonadotropic hypogonadism and menstrual disturbances [6].

We report a rare case of a hormonally active extra‐adrenal paraganglioma in an adolescent presenting with hypertension and secondary amenorrhea, with complete recovery following surgical resection.

## 2 Case Presentation

A 16‐year‐old female was referred for further evaluation of uncontrolled hypertension that had remained refractory for two years despite the use of three antihypertensive medications. She reported a concurrent two‐year history of secondary amenorrhea, which had followed two previously regular menstrual cycles. This was associated with a one‐year history of episodic headaches, palpitations, and excessive sweating. Her father primarily sought medical consultation due to the amenorrhea and the episodic triad of headache, palpitations, and diaphoresis.

Physical examination revealed a thin adolescent with underdeveloped secondary sexual characteristics, consistent with Tanner stage II breast development and sparse axillary and pubic hair. Her blood pressure at presentation was 190/105 mmHg. Her heart rate was recorded at approximately 82 beats per minute (within a range of 70–90 bpm), and there was no evidence of an orthostatic drop in blood pressure during clinical assessment. Abdominal and cervical examinations were unremarkable, with no palpable masses or tenderness noted. At the time of evaluation, the patient had been taking hydrochlorothiazide (25 mg daily), enalapril (10 mg twice daily), and spironolactone (25 mg daily) for two years without significant improvement in her symptoms or blood pressure control.

The patient underwent a systematic evaluation for secondary hypertension. Laboratory reports revealed a mild normocytic, normochromic anemia (hemoglobin 9.5 g/dL), which was clinically categorized as anemia of chronic disease. This was attributed to the underlying pathology and resolved completely following tumor resection. Initial imaging, including an abdominal ultrasound, renal artery Doppler study, and echocardiography, was within normal limits. Other baseline investigations—including renal and liver function tests, serum electrolytes, thyroid function tests, basal cortisol, urinalysis, fasting blood sugar, serum calcium, and viral markers—were negative.

Due to the high clinical suspicion of catecholamine excess, biochemical screening was performed. Results confirmed significant elevations: plasma metanephrine was 334.31 pg/mL (ref: 24–202 pg/mL) and normetanephrine was > 10,000 pg/mL (ref: 14–302 pg/mL). Testing for primary hyperaldosteronism was deferred due to financial constraints. Based on the biochemical evidence and subsequent imaging, the diagnosis of a functional extra‐adrenal paraganglioma was confirmed.

### 2.1 Imaging Findings

Contrast‐enhanced CT imaging demonstrated a well‐defined solid mass measuring 5.4 × 4.8 × 4.4 cm in the left para‐aortic region. The lesion appeared heterogeneously hypoattenuating on non‐contrast images (attenuation was not quantitatively measured). It showed avid enhancement during the arterial phase with subsequent washout on delayed imaging (absolute washout ≈ 55.2%). A focal non‐enhancing area was identified, suggestive of internal necrosis.

The mass demonstrated focal contact with the abdominal aorta; however, the surrounding fat planes were preserved, with no evidence of vascular invasion. No significant retroperitoneal lymphadenopathy was observed, though a few small, likely reactive mesenteric lymph nodes were present.

**FIGURE 1:**
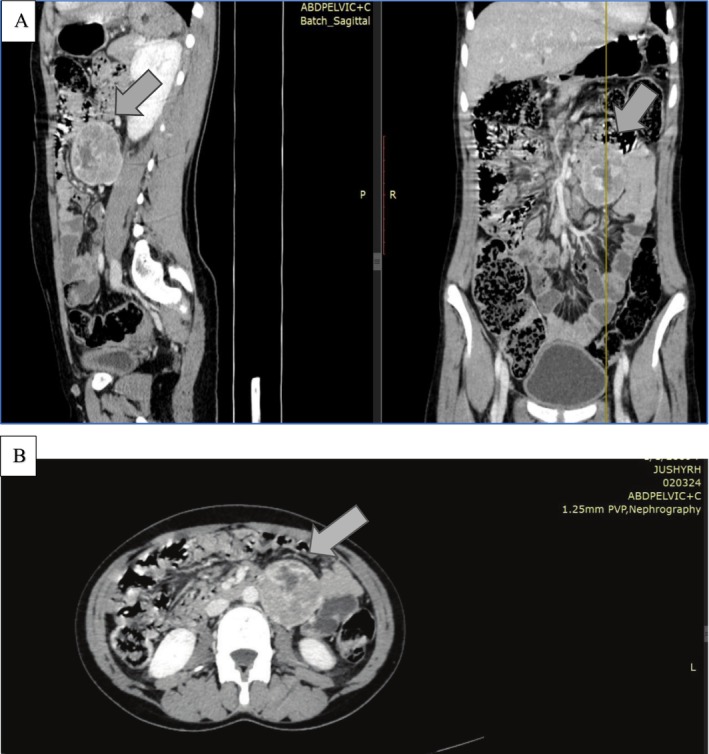
(A) Contrast‐enhanced sagittal and coronal CT views showing a left para‐aortic mass with heterogeneous enhancement and central necrosis. (B) Contrast‐enhanced axial CT view showing the 5.4 × 4.8 × 4.4 cm left para‐aortic mass suggestive of an extra‐adrenal paraganglioma (Organ of Zuckerkandl).

Overall, these imaging features are highly suggestive of an extra‐adrenal paraganglioma, likely arising from the Organ of Zuckerkandl (Figure 1).

### 2.2 Medical Treatment

Following biochemical and imaging confirmation of a functional paraganglioma, the patient's treatment was optimized for surgical preparation. Her existing regimen of enalapril (10 mg twice daily) and spironolactone (25 mg daily) was continued and augmented with alfuzosin (10 mg once daily) to initiate alpha‐blockade. Two weeks later, carvedilol (25 mg twice daily) was added to achieve target hemodynamic stability.

This multi‐drug regimen successfully controlled her blood pressure within the range of 120–135/70–80 mmHg for a total of three months prior to surgery. Despite this sustained period of cardiovascular stability, there was no clinical improvement in her secondary amenorrhea, indicating that medical stabilization was insufficient to reverse the endocrine suppression.

Definitive surgical intervention was delayed during this time due to financial constraints and the necessity of family counseling. The patient was eventually referred to a specialized multidisciplinary team in Addis Ababa—approximately 600 km from Jigjiga—to access expert endocrinologic surgical care. Preoperative volume expansion was achieved via intravenous administration of normal saline (1 L preoperatively and 1 L intraoperatively) to mitigate perioperative hemodynamic instability. No additional oral salt supplementation was administered. The final doses of her antihypertensive medications were administered on the morning of the procedure.

### 2.3 Surgical Intervention

The patient underwent open retroperitoneal tumor resection performed by a multidisciplinary team comprising an endocrinology surgeon, a urology surgeon, an anesthesiologist, and operating room nurses. Despite adequate preoperative blood pressure control over an appropriate duration, the operation was intermittently interrupted due to episodic hypertensive crises, with systolic and diastolic blood pressure rising up to 200/130 mmHg.

**FIGURE 2:**
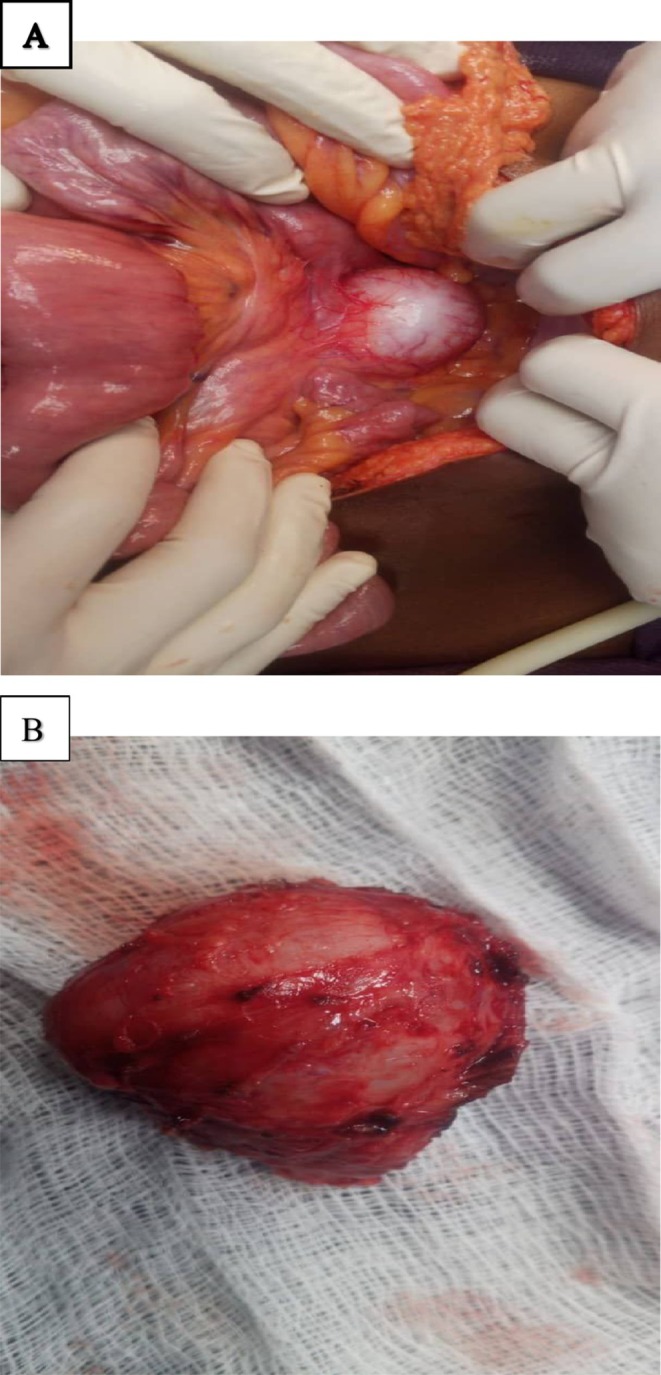
(A) Gross intraoperative photograph of the encapsulated nodular retroperitoneal. (B) Gross photograph of the completely excised encapsulated nodular mass measuring 8 × 6 × 3.5 cm.

These intraoperative hypertensive surges were managed with intravenous phentolamine, administered in two doses (2 mg followed by 3 mg, given 5 min apart), resulting in adequate hemodynamic stabilization. The procedure was then completed successfully without major intraoperative complications, and the tumor was completely excised (Figure 2).

Postoperatively, the patient remained hemodynamically stable, with no episodes of hypotension requiring vasopressor support. All antihypertensive medications were discontinued immediately after surgery, and her blood pressure remained within the normal range during hospitalization and follow‐up.

### 2.4 Pathological Findings

Gross examination revealed a well‐encapsulated, nodular mass measuring 8 × 6 × 3.5 cm. On cut section, the tumor was well‐circumscribed with a characteristic tan‐brown appearance. Histopathological evaluation confirmed a paraganglioma arising in the left para‐aortic retroperitoneal region. Microscopically, the tumor exhibited the classic “Zellballen” architecture, composed of nests of polygonal chief cells with abundant eosinophilic cytoplasm surrounded by delicate sustentacular cells.

**FIGURE 3:**
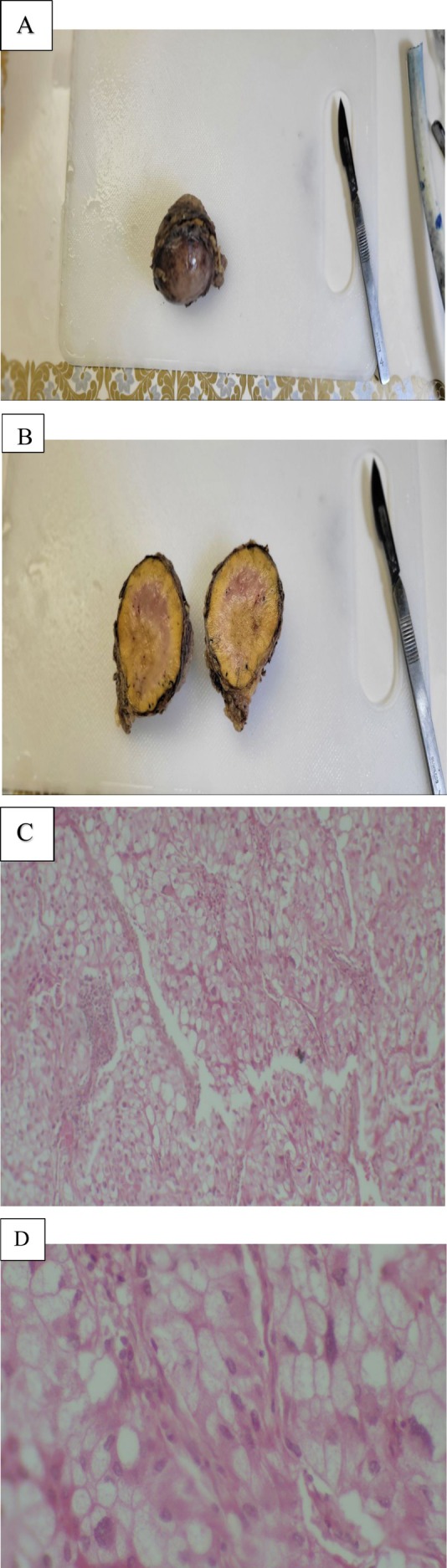
(A) Intact encapsulated nodular mass 8 × 6 × 3.5 cm with a brownish, bosselated surface. (B) Cut section of the tumor showing a tan‐yellow granular interior with a central gray‐brown area (5.5 × 4.5 cm) and an intact capsule. (C) Histopathology (low power) demonstrating nests and trabecular patterns of polygonal cells with granular eosinophilic cytoplasm. (D) Histopathology (high power) confirming the characteristic *Zellballen* pattern with low mitotic activity and no significant cellular atypia.

The tumor showed low mitotic activity and lacked significant cytological atypia or necrosis. The maximum tumor dimension on histopathological assessment was, corresponding to pT2 staging. Ki‐67 immunohistochemical staining was not performed due to institutional resource limitations. However, the PASS score was 3, suggesting a low risk of metastatic behavior (Figure 3).

### 2.5 Postoperative Course and Follow‐Up

All antihypertensive medications were discontinued immediately postoperatively, and her blood pressure remained within the normal range thereafter. Menstruation resumed within one month following surgery after two years of amenorrhea, and hemoglobin increased to 12 g/dL at the one‐month follow‐up.

She was followed monthly for three months, remained asymptomatic, and resumed normal daily activities. Genetic testing was not performed due to financial constraints; however, the possibility of a syndromic association was discussed with the patient and her family. She was advised to have lifelong surveillance, including clinical and biochemical evaluation once or twice yearly, to monitor for tumor recurrence or the development of new lesions.

In conclusion, this is a 16‐year‐old girl who presented with hypertension, secondary amenorrhea, and episodic headaches, sweating, and palpitations for about two years. She was treated with antihypertensive medications but had a poor response. A diagnosis of paraganglioma was later established following biochemical and imaging confirmation after evaluation by a nephrologist. Her blood pressure was well controlled for three months prior to surgery.

The surgery was performed by a multidisciplinary team, including an endocrine surgeon, urosurgeon, anesthesiologist, and other team members. The procedure was uneventful except for an intraoperative hypertensive crisis, which was managed safely.

Following surgery, she did not experience any postoperative or hormone‐related complications. Her blood pressure has remained within the normal range since then. The episodic symptoms did not recur, and her menses resumed one month later. Her hemoglobin also improved to the normal range. This case highlights an important endocrinologic–cardiovascular intersection in paraganglioma.

## 3 Discussion

Adolescent paragangliomas are exceedingly rare, particularly in Sub‐Saharan Africa where reported cases remain limited [1, 8]. This case underscores the importance of considering pheochromocytoma–paraganglioma (PPGL) in adolescents presenting with hypertension, even when endocrine manifestations predominate.

While the classical triad of paraganglioma—headache, palpitations, and sweating—is well recognized [2, 9], reproductive endocrine manifestations such as secondary amenorrhea are rarely reported [6]. In this patient, prolonged catecholamine excess from an organ of Zuckerkandl paraganglioma likely disrupted the hypothalamic–pituitary–gonadal axis, resulting in hypogonadotropic hypogonadism. Experimental studies suggest that norepinephrine can suppress GnRH neuron activity and inhibit luteinizing hormone pulsatility, while adrenergic receptor activation in ovarian tissue may impair steroidogenesis [10]. Notably, in this case, endocrine dysfunction preceded the classical triad, highlighting the need for broader clinical suspicion.

The spontaneous resumption of menses and progression of secondary sexual characteristics following tumor resection confirm the reversible nature of catecholamine‐induced reproductive dysfunction [6]. Clinicians should therefore consider secondary causes, including PPGL, when evaluating unexplained amenorrhea in hypertensive adolescents to prevent prolonged endocrine and cardiovascular complications.

**FIGURE 4:**
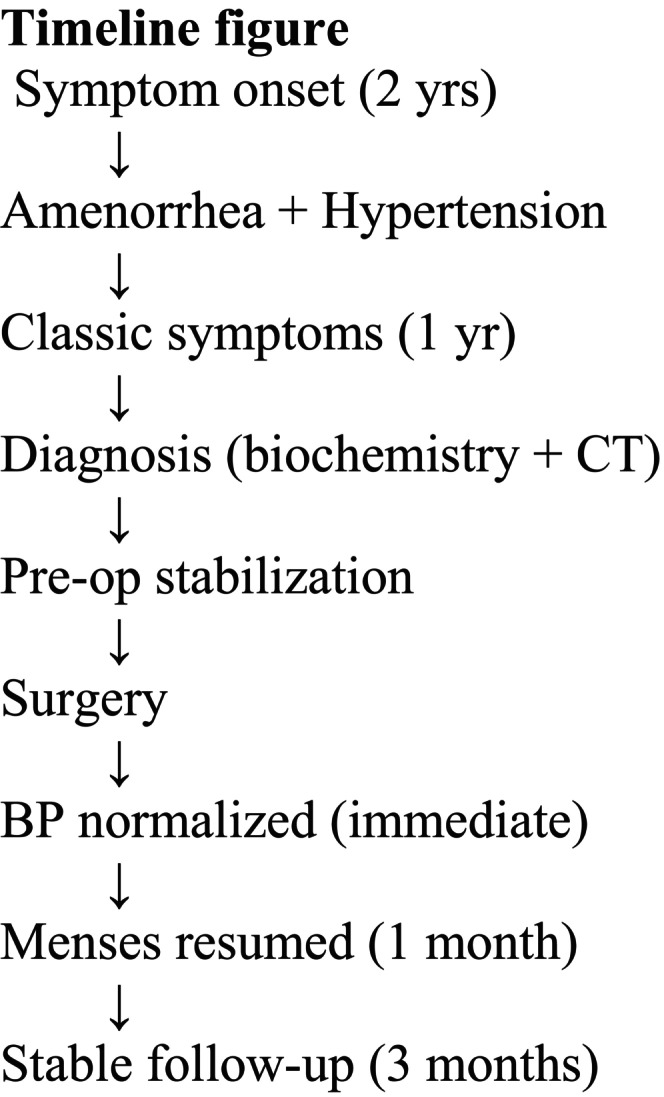
Clinical timeline of disease course and management.

The overall clinical course, including symptom progression, diagnosis, treatment, and postoperative recovery, is summarized in Figure 4.

Diagnosis of PPGL requires biochemical confirmation (plasma‐free metanephrines or urinary catecholamines) alongside imaging [2, 9]. However, limited access to these diagnostic tools in many low‐resource settings can delay diagnosis, as observed in this case. Functional imaging modalities such as MIBG scintigraphy or PET scanning were not performed due to limited availability, representing a diagnostic limitation. Strengthening regional diagnostic capacity is essential to improve early detection and outcomes [8].

Perioperative management of PPGL is complex due to the risk of intraoperative hypertensive crises [1, 9]. In this patient, blood pressure fluctuations during tumor manipulation required intravenous antihypertensives and temporary surgical pauses. Phenoxybenzamine was not used due to limited availability. Instead, preoperative α‐blockade was achieved using alfuzosin, reflecting real‐world practice in resource‐limited settings. Despite these challenges, the favorable outcome highlights the importance of careful perioperative planning, close hemodynamic monitoring, and multidisciplinary care [1, 9].

Complete surgical resection remains the definitive treatment for functional paragangliomas [3, 9]. In this case, normalization of blood pressure without ongoing antihypertensive therapy demonstrates the potential for full cardiovascular recovery following timely intervention [1, 4]. The discrepancy in tumor size between imaging and pathological assessment may reflect differences in measurement techniques and post‐excision tissue handling. Histopathological findings were consistent with a low proliferative tumor profile [3, 5].

Genetic testing is recommended in patients with PPGL diagnosed at a young age, as up to 30%–40% of cases are associated with hereditary syndromes involving SDHx, VHL, RET, or NF1 mutations [7, 9]. However, lack of access to genetic testing in many African settings limits comprehensive risk assessment and family counseling. Expanding access to molecular diagnostics is therefore critical for improving long‐term management [8].

This report has several limitations, including the absence of serum LH, FSH, and estradiol measurements, as well as the lack of functional imaging.

Few reports have described reproductive outcomes following paraganglioma resection in adolescents [6]. The rapid restoration of normal menstruation in this patient highlights the reversible nature of catecholamine‐induced endocrine dysfunction and provides important prognostic insight. This case emphasizes the need for heightened clinical awareness and timely intervention, particularly in resource‐limited settings, where early diagnosis can lead to complete recovery of both cardiovascular and reproductive function [6, 9].

## 4 Conclusion

This case highlights the rare occurrence of extra‐adrenal paraganglioma in an adolescent presenting with secondary amenorrhea and delayed puberty. Early recognition, appropriate biochemical and imaging evaluation, and multidisciplinary management enabled successful tumor resection, normalization of blood pressure, and recovery of reproductive function. Clinicians should consider PPGL in adolescents with hypertension and unexplained endocrine disturbances. Even in resource‐limited settings, timely diagnosis and proper perioperative care can lead to excellent outcomes. Long‐term follow‐up remains essential to monitor recurrence and support overall development.

### 4.1 Strength and Limitation of the Study

This case highlights the importance of a focused etiological evaluation of hypertension rather than solely treating blood pressure as a clinical end point. It also illustrates the complex interaction between catecholamine‐secreting tumors, hypertension, and reproductive health. Furthermore, the case underscores the value of a multidisciplinary, team‐based approach in restoring the patient's overall well‐being and achieving a favorable clinical outcome.

The primary limitations of this report include financial and institutional resource constraints, which precluded a comprehensive hormonal workup both pre‐ and postoperatively. Specifically, LH, FSH, and estradiol levels were not measured to evaluate the patient's secondary amenorrhea, nor was the aldosterone‐to‐renin ratio assessed.

Radiologically, the initial report lacked specific Hounsfield Units (HU) for densitometry, and advanced functional imaging (^123^I‐MIBG or PET) was unavailable to definitively rule out multifocality. Furthermore, Ki‐67 immunohistochemical staining was not performed to assess the tumor's proliferative index. Finally, genetic testing for germline mutations was not conducted, which remains a significant omission given the patient's young age and the extra‐adrenal location of the tumor.

## Author Contributions

**Getasew Kassaw Alemu:** conceptualization, data curation, formal analysis, validation, visualization, writing – original draft, writing – review and editing. **Ibraist Yohannes Haileyesus:** formal analysis, investigation, writing – original draft, writing – review and editing. **Hirsi Haahi Ahmed:** data curation, methodology, writing – review and editing. **Ahmednasir Abdi Hasan:** data curation, methodology, writing – review and editing. **Mubarek Abdi Mohamed:** data curation, methodology, writing – review and editing.

## Funding

The authors have nothing to report.

## Disclosure

The authors have nothing to report.

## Ethics Statement

The author's institution does not require ethical approval for the publication of a single case report.

## Consent

The patient provided written informed consent for the publication of this case report, including clinical history, laboratory data, and imaging findings.

## Data Availability

Data sharing is not applicable to this article as no novel datasets were generated or analyzed during the current study. All relevant clinical findings, imaging, and histopathological data supporting the conclusions of this case report are fully disclosed within the manuscript text and its associated figures.
